# Supplementary material for: A computational method for immune repertoire mining that identifies novel binders from different clonotypes, demonstrated by identifying anti-pertussis toxoid antibodies
Source: MAbs. 2021 Jan 11;13(1):1869406. doi: 10.1080/19420862.2020.1869406 (PMC7808390; doi:10.1080/19420862.2020.1869406)
Supplement: Supplemental Material [file KMAB_A_1869406_SM0620.zip › SUPPLEMENT/Supplementary_material_clean.docx]

**A computational method for immune repertoire mining that identifies novel binders from different clonotypes, demonstrated identifying anti-Pertussis toxoid antibodies**

Supplementary information

Eve Richardson1, Jacob D. Galson2,3, Paul Kellam4,5, Dominic F. Kelly6,7, Sarah E.

Smith4, Anne Palser4, Simon Watson4, and Charlotte M. Deane*1

1Department of Statistics, University of Oxford, UK

2Alchemab Therapeutics Ltd, London, UK

3University Children’s Hospital, University of Zurich, Switzerland

4Kymab Ltd, Cambridge, UK

5Department of Infectious Disease, University College London, UK

6Department of Paediatrics, University of Oxford, UK

7Oxford University Hospitals NHS Foundation Trust, Oxford, UK

*To whom correspondence should be addressed; deane@stats.ox.ac.uk

| Example | PDB | VH/JH/H1/H2/H3 | VL/JL/L1/L2/L3 |
| --- | --- | --- | --- |
| 1 | 4zpv_HL | IGHV9-5-3/IGHJ4/AASGFTFSSYAMS/TISSGGTYTY/  VRDGNSMDY | IGKV10-96/IGKJ5/ASQDINNYLN/YYTSRLHS/QQANTLPPT |
| 1 | 5do2_HL | IGHV5-6-4/IGHJ2/AASGFTFSSYTMS/TISSGGSYTY/TRDGNDYDY | IGKV10-96/IGKJ1/ASQDISNYLN/YYTSRLHS/QQGNTLPRT |
| 2 | 1c08_BA | IGHV3-8/IGHJ3/SVTGDSITSDYWS/YVSYSGSTY/ANWDGDY | IGKV5-43/IGKJ2/ASQSIGNNLH/KYASQSIS/QQSNSWPYT |
| 2 | 1dqj_BA | IGHV3-8/IGHJ1/SVTGDSVTSDYWS/YISYSGSTY/ASWGGDV | IGKV5-43/IGKJ2/ASQSISNNLH/KYASQSIS/QQSNSWPYT |

Table 1: V and J genes and CDRs of the VHs and VLs of the pairs of antibodies binding to the same epitope in Figure 1 (main text) (Example 1) and Supplementary Figure 2 (Example 2). In example 1, two murine antibodies with different VH, JH and JK genes bind to the same epitope on the MERS-CoV spike protein. In example 2, two murine antibodies bind to the same epitope on lysozyme have differing IGHJ genes. In both instances, the pairs of sequences would be separated by standard clonotyping definitions.

| **ID** | **Framework** | **CDRH1** | **CDRH2** | **CDRH3** |
| --- | --- | --- | --- | --- |
| **CL-97155** | 3k2u | 5uea | 6elu | 1iqd |
| **CL-97116** | 3k2u | 5uea | 6elu | 1iqd |

Table 2: PDB IDs of templates used in homology modelling of a pair of antibodies with just 40% CDRH3 amino acid identity but 80% predicted paratope identity, as referred to in the ’Paratyping and clonotyping successfully cluster PTx binders in a single-cell dataset’ section of Results. Sequences were homology modelled using ABodyBuilder [1] which assigns structural templates to each of the CDRs and the framework region. Common template usage across all of the CDRs and framework region indicates structural similarity (structural similarity despite low sequence identity is noted elsewhere [2]). The relationship between these two Pertussis-toxoid binding antibodies was recovered through paratyping and demonstrates that low sequence identity hits can have both chemically and structurally similar binding sites.


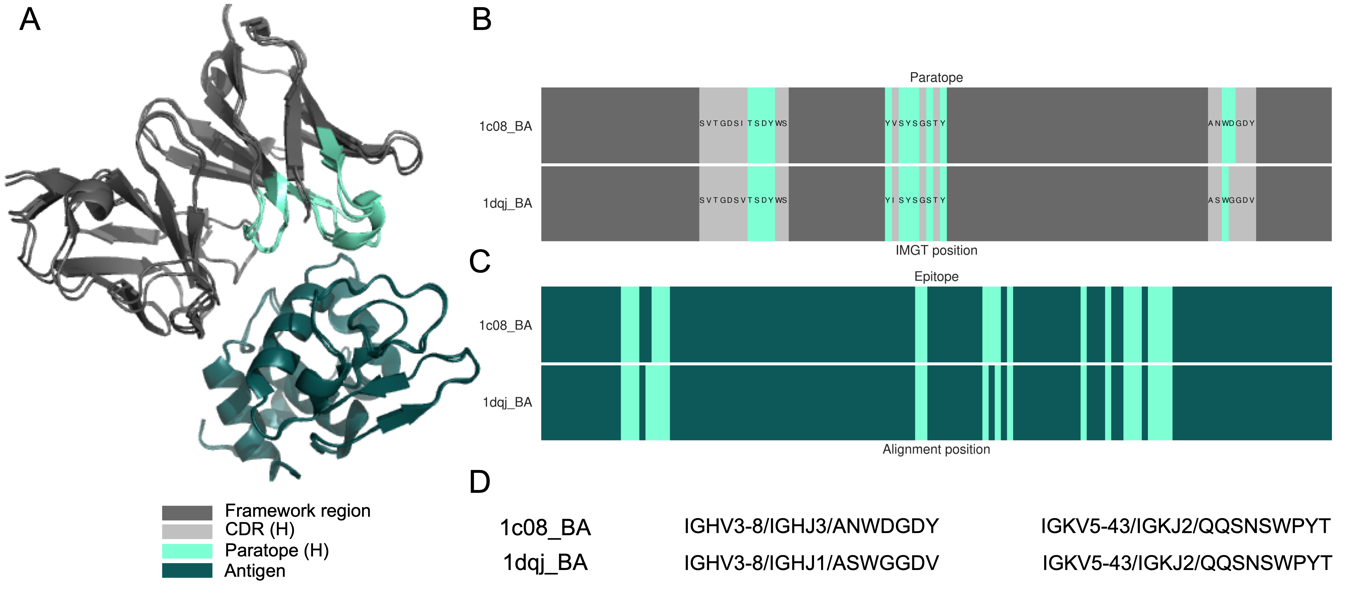


Figure 1: An example of two antibodies that bind to the same epitope but derive from different clonal lineages. HyHel-10 (PDB ID: 1c08) and HyHel-63 (PDB ID: 1dqj) anti-hen egg white lysozyme antibodies target the same residues, despite being derived from different J genes and displaying CDRH3 amino acid identity (57.1%) below the standard clonotyping definition (80% - 100%) (C). The antibodies use the same paratope residues (100% paratope identity) (B) to achieve this functional convergence (95.7% epitope identity). The paratope and epitope are defined as those residues with any atom within 4.5 ˚A of any residue in the antigen or antibody respectively. Epitope and paratope identity correspond to sequence identity at equivalent epitope and paratope residues in the antigen and antibody alignments respectively, where the denominator is the minimum number of epitope or paratope residues in the pair of structures being compared.


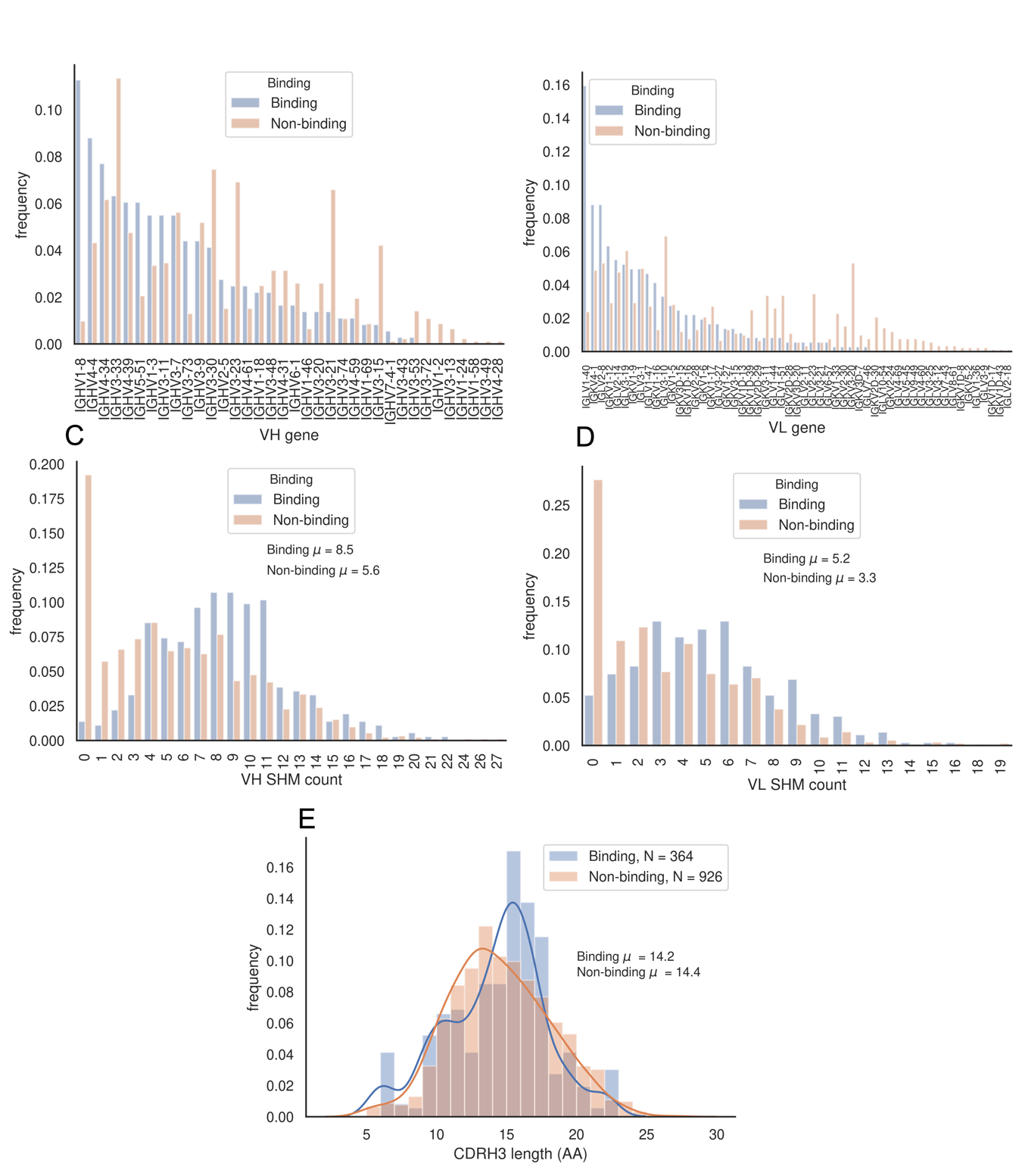


Figure 2: (A) VH frequencies in the binding and non-binding populations of antibodies from the single-cell experiment (N = 1290, of which 364 are binding and 926 non-binding). Over 10% of PTx-binding antibodies are derived from IGHV1-8. (B) VL frequencies in the binding and non-binding antibodies. The most common light chain in binding antibodies is IGLV1-40. Somatic hypermutation (SHM) counts in across the VH (C) and VL (D) counts reveal that the non-binding antibodies have on average lower SHM counts across both VH and VL chains. (E) The CDRH3 length distributions in the binding and non-binding antibodies are similar, with an average length of 14 amino acids.

similar, with an average length of 14 amino acids.

| Category | Probe information | New binder information | CDRH3 identity |
| --- | --- | --- | --- |
| P | IGHV3-23/IGHJ6/AKGGAVSFYYYFGMDV | IGHV3-23/IGHJ6/AKHSSSWFYYYYGMVV | 0.562 |
| P | IGHV5-51/IGHJ6/ARQKSGNYYNGMDV | IGHV5-51/IGHJ4/ARLRSGSYYNAFDY | 0.571 |
| P | IGHV4-59/IGHJ3/TREITGNAFDI | IGHV4-59/IGHJ3/GRERIGEAFDI | 0.636 |
| P | IGHV3-9/IGHJ6/AKHMSRIPVAGSGGMDV | IGHV3-9/IGHJ6/TKDMSRIAVAGTCGMDV | 0.706 |
| P | IGHV5-51/IGHJ6/ARQKSGNYYNGMDV | IGHV5-51/IGHJ6/ARQKIGNFYNAIDV | 0.714 |
| C | IGHV4-59/IGHJ3/TREITGNAFDI | IGHV4-59/IGHJ3/ARERTGEAFDI | 0.727 |
| P | IGHV4-39/IGHJ4/ARGLLMWFGESHFDY | IGHV4-39/IGHJ6/ARGVLMWFGESGMDV | 0.733 |
| C | IGHV3-33/IGHJ6/SRDLVATIYYYGMDV | IGHV3-33/IGHJ6/ARDLGATHYYFGMDV | 0.733 |
| C | IGHV4-39/IGHJ4/ARGLLMWFGESHFDY | IGHV4-39/IGHJ4/ARGILLWFGESSFDH | 0.733 |
| C | IGHV3-23/IGHJ6/AKGGAVSFYYYFGMDV | IGHV3-23/IGHJ6/AKGGAAAYYYYYGMDV | 0.75 |
| C | IGHV3-33/IGHJ6/ARERIIRGIMDV | IGHV3-33/IGHJ6/AREKAIRGLMDV | 0.75 |
| C | IGHV3-11/IGHJ2/AREGFYDVLTDYYRNWYFDL | IGHV3-11/IGHJ2/AREGHHDILTGYYRYWYFDL | 0.75 |
| C | IGHV3-7/IGHJ6/ARDWVKVRGLTGYRYYGLDV | IGHV3-7/IGHJ6/ARDWIKVRRLIGYYYYGMDV | 0.75 |
| C | IGHV3-33/IGHJ5/AREFYDISTGYSNWFDP | IGHV3-33/IGHJ5/AREYFDVSTGFSNWFDP | 0.765 |
| B | IGHV6-1/IGHJ5/SKESGIYQGWFDP | IGHV6-1/IGHJ5/ATESGSYQGWFDP | 0.769 |
| P | IGHV4-39/IGHJ4/ARGVLLWFGESSFDY | IGHV4-39/IGHJ6/ARGVLLWFGESGMDV | 0.8 |
| B | IGHV3-11/IGHJ6/AREYSNNWDYYVMDV | IGHV3-11/IGHJ6/AREYSSRWDYYGMDV | 0.8 |
| B | IGHV3-73/IGHJ6/SIPGADGMDV | IGHV3-73/IGHJ6/SRPGTDGMDV | 0.8 |
| C | IGHV3-33/IGHJ6/AREPFYDILTNYYYYYYYGMDV | IGHV3-33/IGHJ6/ARHPHYDVLTNYYYYYYYGLDV | 0.818 |
| C | IGHV3-33/IGHJ5/AREFYDISTGYSNWFDP | IGHV3-33/IGHJ5/AREYNDISTGYSSWFDP | 0.824 |
| C | IGHV4-4/IGHJ4/ARDYYGSGSYFDY | IGHV4-4/IGHJ4/ARTYYGSGTYFDY | 0.846 |
| C | IGHV3-7/IGHJ6/ARDWVMVRGLIGYYYYGMDV | IGHV3-7/IGHJ6/ARDWVKVRGRIGFYYYGMDV | 0.85 |
| C | IGHV3-43/IGHJ6/AKNYYGSGSYYDYYYYYGMDV | IGHV3-43/IGHJ6/AKNYFGSGNYEDYYYYYGMDV | 0.857 |
| B | IGHV4-34/IGHJ6/TREGWFGESLGLDV | IGHV4-34/IGHJ6/AREGWFGESLGMDV | 0.857 |
| C | IGHV5-51/IGHJ6/ARQKSGNYYNGMDV | IGHV5-51/IGHJ6/ARQKIGNYYHGMDV | 0.857 |
| C | IGHV3-74/IGHJ6/ARDPYYDILTRYYYYYYYGMDV | IGHV3-74/IGHJ6/ARDPHYDIMTNYYYYYYYGMDV | 0.864 |
| P | IGHV4-34/IGHJ4/ARENYDILTGSHFDY | IGHV4-59/IGHJ4/AREDYDILTGSYFDY | 0.867 |
| P | IGHV4-34/IGHJ4/ARENYDILTGSHFDY | IGHV4-59/IGHJ4/AREGYDILTGSYFDY | 0.867 |
| B | IGHV1-8/IGHJ6/ARDGRMDV | IGHV1-8/IGHJ6/ARDSRMDV | 0.875 |
| C | IGHV3-33/IGHJ4/ARDHDFLTGYSSRFDY | IGHV3-33/IGHJ4/ARDHDIMTGYSSRFDY | 0.875 |
| C | IGHV3-33/IGHJ5/AREFYDISTGYSNWFDP | IGHV3-33/IGHJ5/AREYNDISTGYSNWFDP | 0.882 |
| B | IGHV3-9/IGHJ6/AKHMSRIPVAGSGGMDV | IGHV3-9/IGHJ6/AKHMSRTPVAGAGGMDV | 0.882 |
| C | IGHV3-33/IGHJ5/AREFYDISTGYSNWFDP | IGHV3-33/IGHJ5/AREYYDVSTGYSNWFDP | 0.882 |
| C | IGHV3-48/IGHJ4/ARGAPYFYGWGSYYFDY | IGHV3-48/IGHJ4/ARGAPYYYGSGSYYFDY | 0.882 |
| C | IGHV1-3/IGHJ4/ARDNLWFDY | IGHV1-3/IGHJ4/ARDNLSFDY | 0.889 |
| B | IGHV1-3/IGHJ4/ARDKWEILDY | IGHV1-3/IGHJ4/ARDKWELLDY | 0.9 |
| B | IGHV3-7/IGHJ6/ARDWVMVRGLIGYYYYGMDV | IGHV3-7/IGHJ6/ARDWVKVRGLIGHYYYGMDV | 0.9 |
| B | IGHV1-3/IGHJ4/ARDYFGSGNY | IGHV1-3/IGHJ4/ARDYFGSGSY | 0.9 |
| B | IGHV3-73/IGHJ6/TIPGTDGMDV | IGHV3-73/IGHJ6/TRPGTDGMDV | 0.9 |
| B | IGHV3-74/IGHJ6/ARDPYYDILTRYYYYYYYGMDV | IGHV3-74/IGHJ6/ARDPYYDILTNYYYYYYYGMDA | 0.909 |
| B | IGHV1-8/IGHJ4/ARRGYSYGYDY | IGHV1-8/IGHJ4/ARRGYSYGYDF | 0.909 |
| C | IGHV3-74/IGHJ6/ARDPYYDILTRYYYYYYYGMDV | IGHV3-74/IGHJ6/ARDPHYDILTNYYYYYYYGMDV | 0.909 |
| B | IGHV4-39/IGHJ4/ARQEVREIIFDY | IGHV4-39/IGHJ4/ARQEVREIFFDY | 0.917 |
| B | IGHV5-51/IGHJ3/ARLLSGDGDAFDF | IGHV5-51/IGHJ3/ARLLSGDGDAFDI | 0.923 |
| B | IGHV3-11/IGHJ2/VREWVRFSYWYFDL | IGHV3-11/IGHJ2/AREWVRFSYWYFDL | 0.929 |
| B | IGHV3-73/IGHJ4/TIHGDILTDYYKDY | IGHV3-73/IGHJ4/TIHGDILTGYYKDY | 0.929 |
| C | IGHV4-39/IGHJ4/ARGVLLWFGESSFDY | IGHV4-39/IGHJ4/ARGVLLWFGESHFDY | 0.933 |
| C | IGHV3-48/IGHJ6/ARDTRYFDWLLDYGMDV | IGHV3-48/IGHJ6/ARDTRYFDWLLEYGMDV | 0.941 |
| B | IGHV3-33/IGHJ5/AREFYDISTGYSNWFDP | IGHV3-33/IGHJ5/AREFFDISTGYSNWFDP | 0.941 |
| B | IGHV3-33/IGHJ5/ARDYYDISTGYSHWFDP | IGHV3-33/IGHJ5/ARDFYDISTGYSHWFDP | 0.941 |
| B | IGHV3-23/IGHJ6/AKDRIVGATNYFYGMDV | IGHV3-23/IGHJ6/AKDRIVGATNYYYGMDV | 0.941 |
| B | IGHV4-34/IGHJ6/AREGMITFGGVIDHYGMDV | IGHV4-34/IGHJ6/AREGMITFGGVIDYYGMDV | 0.947 |

Table 3: V gene, J gene and CDRH3s of the novel PTx binding antibodies and the probe antibodies by which they were identified, where the CDRH3 differs between the probe and the new antibody (66/84 possible confirmed binders). The 52 unique V/J/CDRH3 combinations among those 66 antibodies are listed. Prediction categories are listed as P for paratype-unique (within the paratype of the probe in question; not within the clonotype of any probe antibody), C for clonotype-unique (within the clonotype of the probe in question; not within the paratype of any probe antibody) or B for both (within the paratype and clonotype of the probe antibody in question).


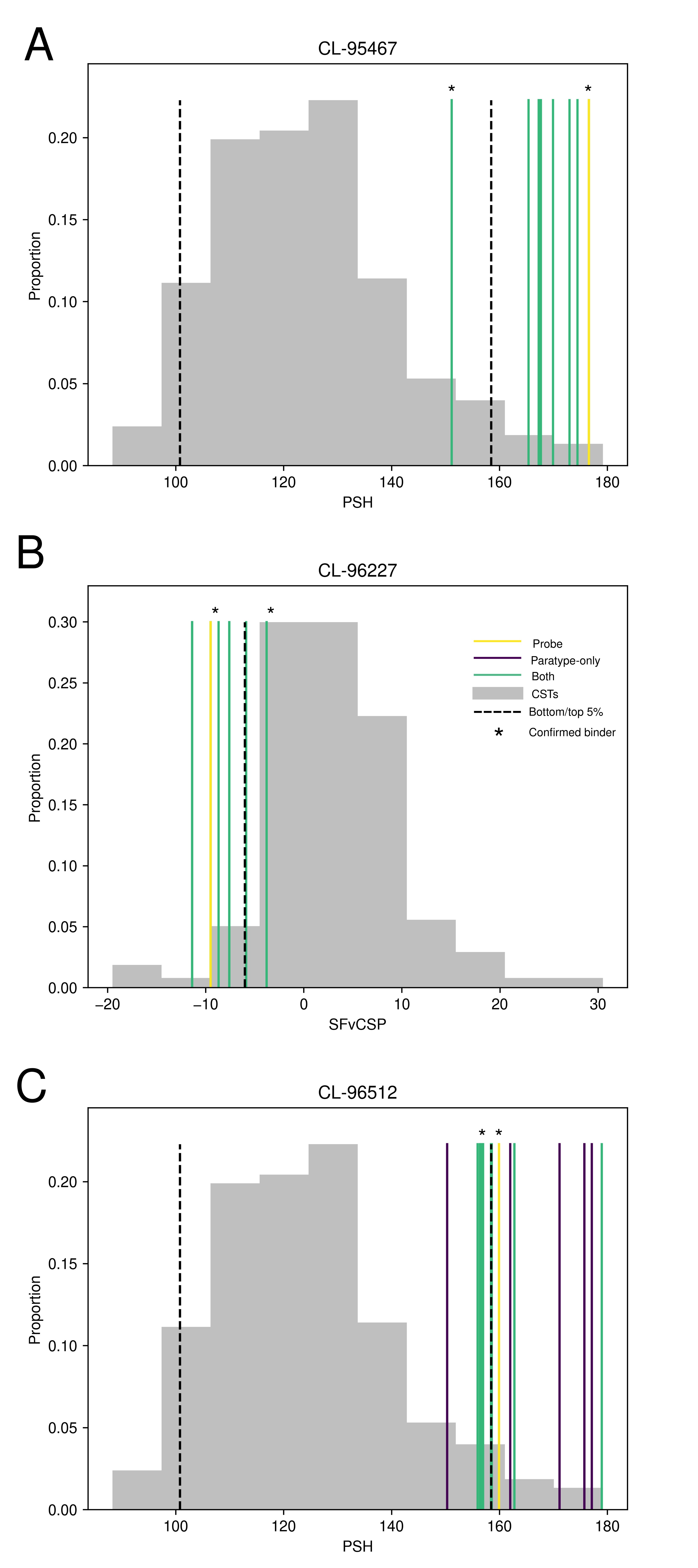


Figure 3: As referred to in the ’Repertoire mining can improve in-silico developability metrics’ section of Results, three further examples of probe antibodies with flagged developability issues (in figures A and C, patch surface hydrophobicity (PSH); in figure B, charge asymmetry between the heavy and light chains (SFvCSP)) where the developability flag was removed in putative PTx binders identified by immune repertoire mining. Asterisks indicate confirmed PTx-binding antibodies, the probe antibody (yellow) and binders identified through prospective experimentation. The putative PTx-binding antibodies may identified by both clonotyping and paratyping (green), paratyping only (purple) or clonotyping only (blue). In these three instances, the change in the property is significant enough that the identified PTx-binding antibody discovered through repertoire mining is no longer flagged. Developability flags are assigned using the Therapeutic Antibody Profiler, TAP [3], which calculates five metrics associated with developability from homology models and compares these values with the distribution observed among clinical-stage therapeutics (CSTs); antibodies that lie in the extremes of a particular metric are ”flagged” with respect to that metric.


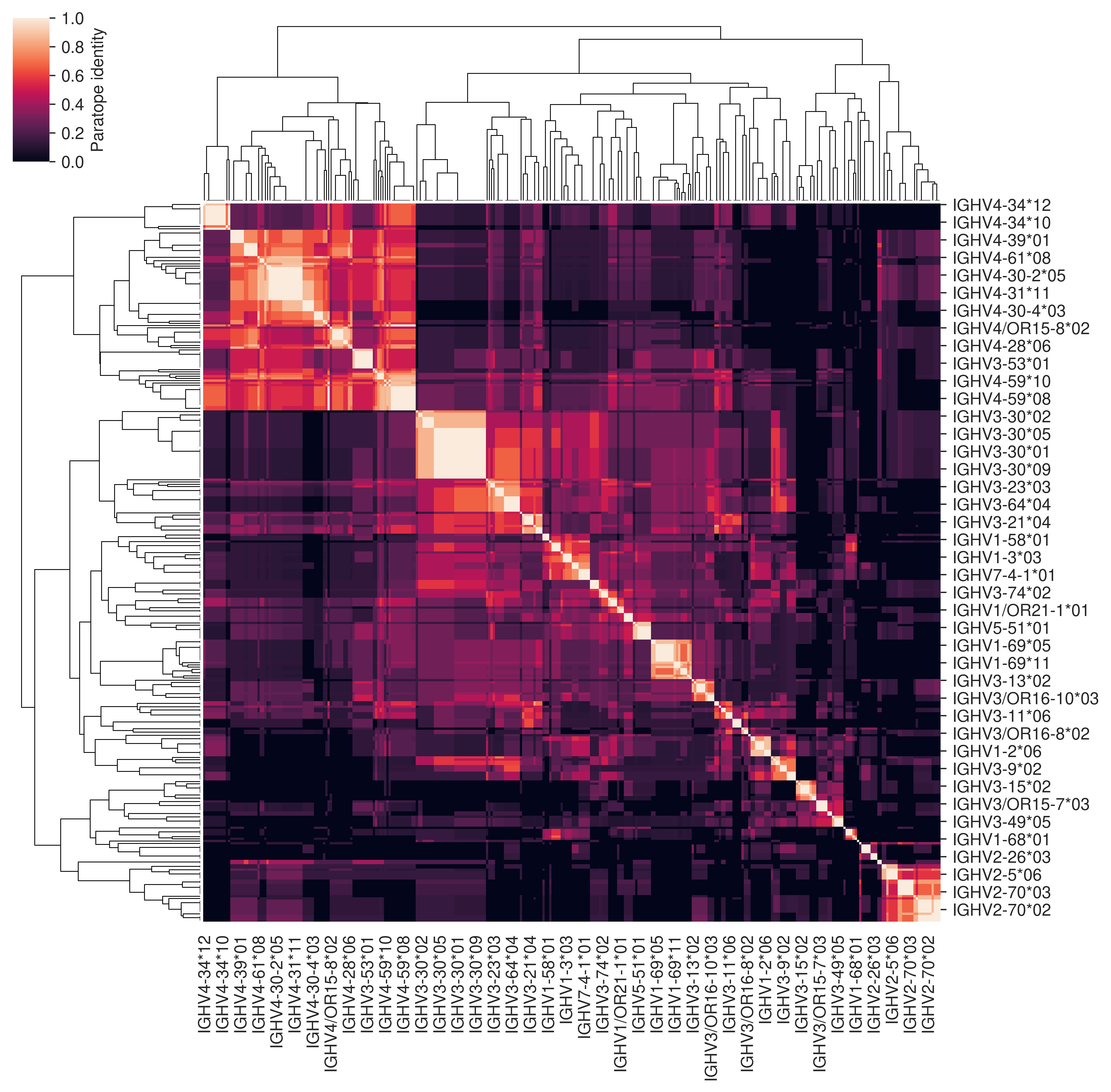


Figure 4: Predicted paratope identity across CDRH1 and CDRH2 of germline alleles (IMGT, as of February 14th 2020). Paratope identity may be equal to or in excess of 75% across V genes within the same and different families - this was detected in alleles from genes IGHV3-53 and IGHV4-59, IGHV3-66 and IGHV4-59 and IGHV7-4-1 and IGHV1-8, suggesting that in large enough sequencing data sets paratyping could cluster antibodies from different V gene families.

| Method | Precision | Recall | F score | Homology model required |
| --- | --- | --- | --- | --- |
| Parapred | 64.5 | 77.2 | 0.70 | No |
| proABC-2 | 65.8 | 64.7 | 0.65 | No |
| iPatch | 42.0 | 89.6 | 0.57 | Yes |

# Table 4: precision, recall and F-score at the optimal thresholds reported in the original papers for different paratope prediction methods on a set of 552 non-redundant (<95% CDRH or CDRL identity) antibody/antigen complex structures (0.67 for Parapred [4], 0.40 for proABC-2 [5], and for iPatch [6], the optimum threshold was chosen as 0.61 using Youden’s J-statistic).

| Paratope prediction method | Precision | Recall |
| --- | --- | --- |
| iPatch | 79 | 76 |
| ProABC-2 | 82 | 74 |
| Parapred | 84 | 73 |

Table 5: Precision-recall values for the single-cell experiment repeated with alternative paratope prediction methods, the structure-based iPatch predictor and sequence-based predictor ProABC-2. Precision and recall are reported at a threshold of 75% predicted paratope identity. The behaviour of the precision-recall curve is stable across paratope prediction methods.

| Antigen | # antibodies | # possible true positives | # paratype predictions | # paratype true positives | Minimum epitope identity (%) |
| --- | --- | --- | --- | --- | --- |
| Hemagglutinin | 79 | 84 | 11 | 8 | 23.1 |
| gp120 | 77 | 132 | 16 | 7 | 16.7 |
| SARS-CoV-2 spike protein | 44 | 21 | 2 | 2 | 58.3 |
| gp160 | 39 | 98 | 33 | 30 | 33.3 |
| Lysozyme | 33 | 221 | 144 | 144 | 53.8 |

Table 6: evaluation of the hypothesis that paratyping groups antibodies which bind to the same epitope on five antigens from SAbDab (October 2020) [7]. For each antigen, the dataset of antibody/antigen structures is made non-redundant at the level of 100% sequence identity across the heavy chain CDRs. Epitope similarity for each pair of antibodies binding to the same antigen is evaluated using Ab-Ligity [8], a stringent definition considering physicochemical and structural similarity. True positives are pairs of antibodies with an Ab-Ligity epitope score greater than or equal to 0.1. The heavy chain predicted paratope of each pair of antibodies with the same length CDRHs is compared. As per the paratype definition, antibodies with greater than 75% predicted paratope identity are within the same paratype and predicted to bind to the same epitope. All pairs of antibodies predicted to bind to the same epitope share epitope residues (minimally 16.7%) and could be argued to be at the same epitope. However, using the Ab-Ligity definition there is a false positive rate between 0% (SARS-CoV-2 spike, lysozyme) and 56.3% (gp120).

# References

1. Raybould, M. I. J. *et al.* Five computational developability guidelines for therapeutic antibody profiling. en. *Proceedings of the National Academy of Sciences* **116,** 4025–4030. issn: 0027-8424, 1091-6490. https://www.pnas.org/content/116/10/4025 (Mar. 2019).
2. Leem, J., Dunbar, J., Georges, G., Shi, J. & Deane, C. M. ABodyBuilder: Automated antibody structure prediction with data–driven accuracy estimation. *mAbs* **8,** 1259–1268. issn: 1942-0862.

https://doi.org/10.1080/19420862.2016.1205773 (Oct. 2016).

1. Raybould, M. I. J. *et al.* Evidence of Antibody Repertoire Functional Convergence through Public Baseline and Shared Response Structures. en. *bioRxiv,* 2020.03.17.993444. <https://www>. biorxiv.org/content/10.1101/2020.03.17.993444v1 (Mar. 2020).
2. Liberis, E., Velickoviˇ c, P., Sormanni, P., Vendruscolo, M. & Li´ o, P. Parapred: antibody paratope` prediction using convolutional and recurrent neural networks. en. *Bioinformatics* **34,** 2944–2950. issn: 1367-4803. https://academic.oup.com/bioinformatics/article/34/17/2944/4972995 (Sept. 2018).
3. Ambrosetti, F. *et al.* proABC-2: PRediction of AntiBody contacts v2 and its application to informationdriven docking. en. *Bioinformatics.* https://academic.oup.com/bioinformatics/advancearticle/doi/10.1093/bioinformatics/btaa644/5873593 (2020) (July 2020).
4. Krawczyk, K., Baker, T., Shi, J. & Deane, C. M. Antibody i-Patch prediction of the antibody binding site improves rigid local antibody–antigen docking. en. *Protein Engineering, Design and Selection* **26.** Publisher: Oxford Academic, 621–629. issn: 1741-0126. https://academic.oup.com/peds/ article/26/10/621/1512673 (2020) (Oct. 2013).
5. Dunbar, J. *et al.* SAbDab: the structural antibody database. en. *Nucleic Acids Research* **42,** D1140– D1146. issn: 0305-1048. https://academic.oup.com/nar/article/42/D1/D1140/1044118 (Jan. 2014).
6. Wong, W. K. *et al.* Ab-Ligity: Identifying sequence-dissimilar antibodies that bind to the same epitope. en. *bioRxiv,* 2020.03.24.004051. https://www.biorxiv.org/content/10.1101/2020.

03.24.004051v1 (Mar. 2020).
