# Supplementary figures and images for: A computational method for immune repertoire mining that identifies novel binders from different clonotypes, demonstrated by identifying anti-pertussis toxoid antibodies
Source: MAbs. 2021 Jan 11;13(1):1869406. doi: 10.1080/19420862.2020.1869406 (PMC7808390; doi:10.1080/19420862.2020.1869406)

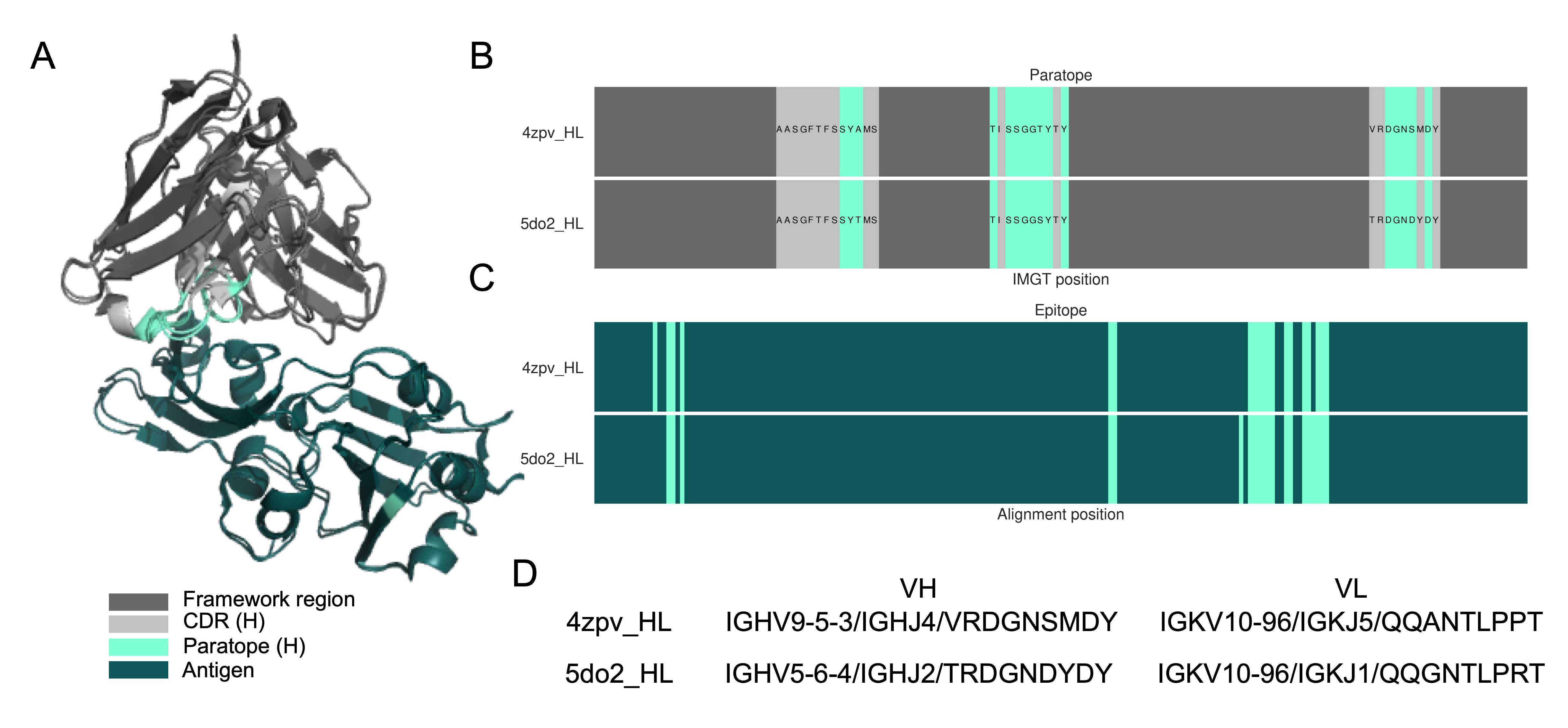

Supplement: Supplemental Material [file KMAB_A_1869406_SM0620.zip › SUPPLEMENT/supplementary_figure1.png]

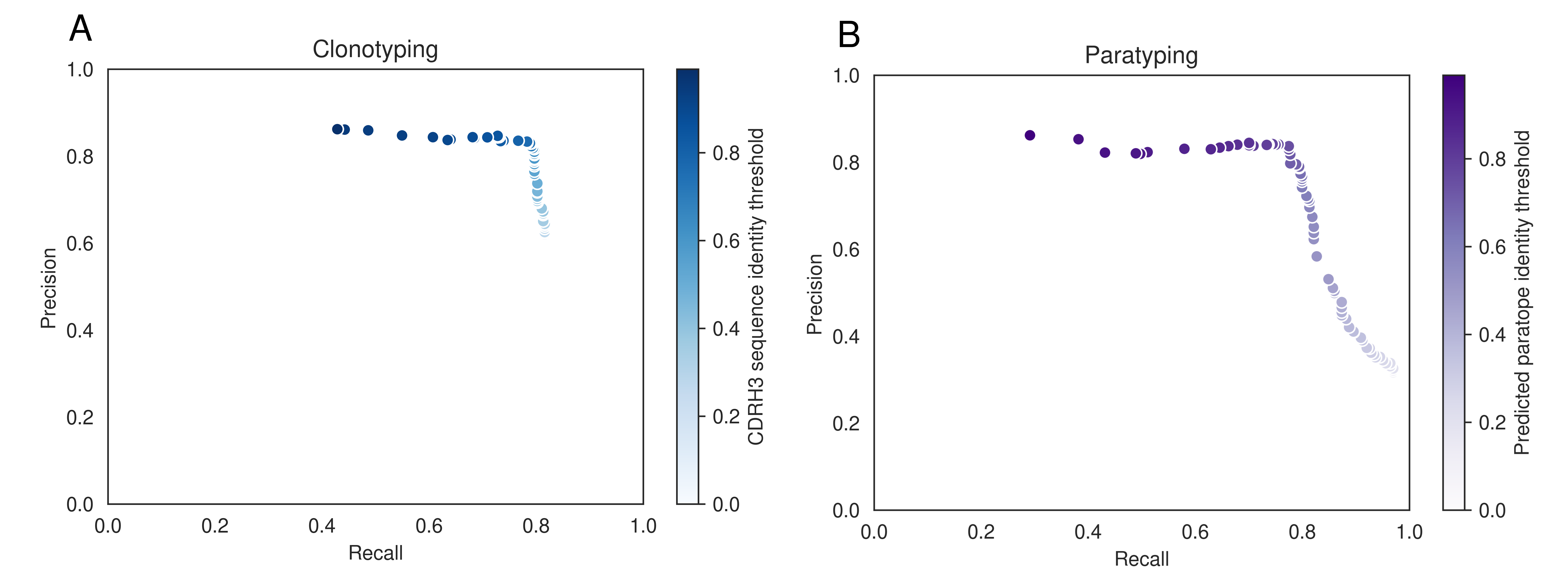

Supplement: Supplemental Material [file KMAB_A_1869406_SM0620.zip › SUPPLEMENT/supplementary_figure2.png]

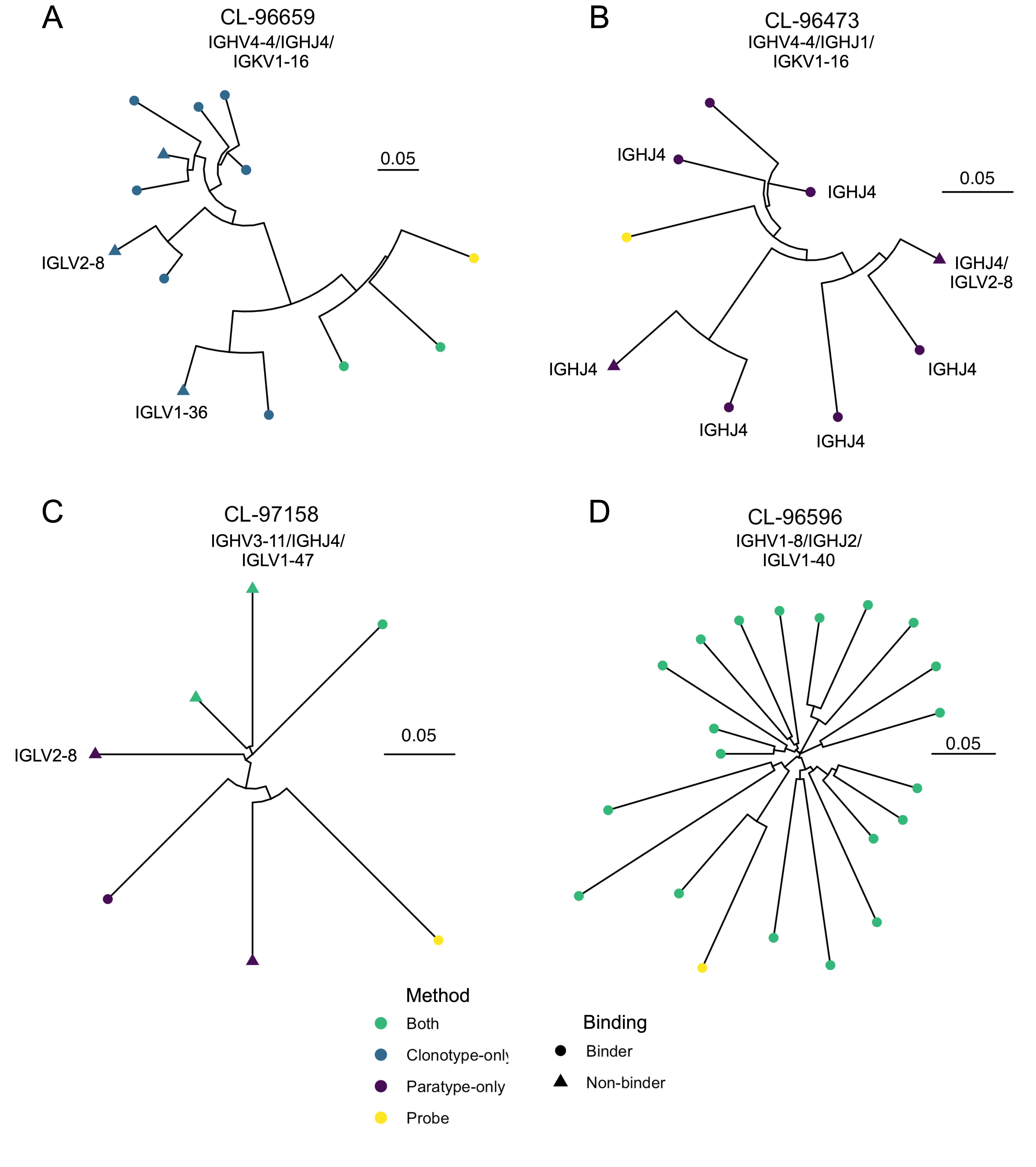

Supplement: Supplemental Material [file KMAB_A_1869406_SM0620.zip › SUPPLEMENT/supplementary_figure3.png]

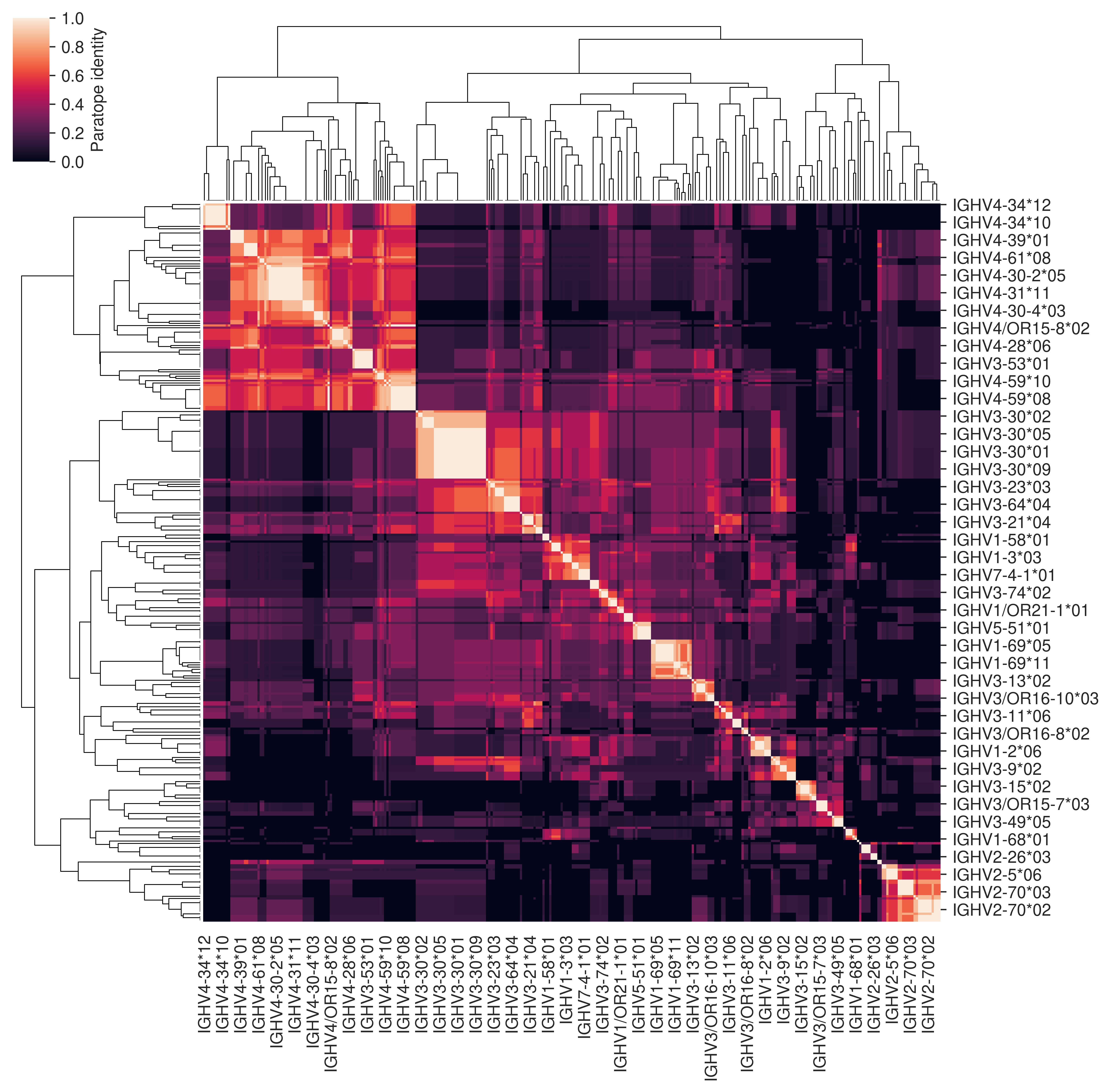

Supplement: Supplemental Material [file KMAB_A_1869406_SM0620.zip › SUPPLEMENT/supplementary_figure4.png]
